# Endogenous Apelin Is Protective Against Age-Associated Loss of Retinal Ganglion Cells in Mice

**DOI:** 10.3389/fnagi.2020.00058

**Published:** 2020-03-20

**Authors:** Yuki Ishimaru, Akihide Sumino, Fumiya Shibagaki, Akiko Yamamuro, Yasuhiro Yoshioka, Sadaaki Maeda

**Affiliations:** ^1^Laboratory of Pharmacotherapeutics, Faculty of Pharmaceutical Sciences, Setsunan University, Hirakata, Japan; ^2^Laboratory of Food Chemistry, Yokohama University of Pharmacy, Yokohama, Japan

**Keywords:** retinal ganglion cell (RGC), apelin, aging, knock-out, cell loss, apelin receptor (APJ)

## Abstract

Age-associated loss of retinal ganglion cells (RGCs) causes visual deficits, but there is not yet any therapeutic agent to prevent the loss of these cells. Herein, we report that apelin, an endogenous peptide ligand of APJ receptor, is protective against the age-related loss of RGCs in mice. The mRNA expression of apelin was reduced in the retina of old mice compared with that in young mice, whereas retinal APJ expression increased with age. Immunofluorescence staining showed that APJ was present in RGCs and their surrounding cells expressed apelin. In addition, both functional and histological analyses demonstrated that apelin deficiency accelerated the loss of RGCs associated with age in mice. These results suggest that endogenous apelin plays a protective role against the degeneration of RGCs and that the apelinergic axis may be a new target for preventing age-related visual impairment.

## Introduction

Age-related visual impairments can occur even in the absence of recognized eye diseases (Trick, 1987; Langrová et al., 2008). Retinal ganglion cells (RGCs) are neurons that transmit visual information from the retina to the brain and are more vulnerable to age-related loss than other retinal neurons (Cavallotti et al., 2001; Neufeld and Gachie, 2003). Therefore, the identification of protective factors against RGC loss with age should aid in the development of drugs that prevent visual deficits in the elderly.

Apelin is an endogenous peptide ligand of the G protein-coupled receptor APJ (Tatemoto et al., 1998). APJ stimulation by apelin in neurons results in activation of pro-survival signaling pathways that can afford neuroprotection (O’Donnell et al., 2007; Zeng et al., 2010; Cook et al., 2011). We previously reported that apelin deficiency in mice accelerated the loss of motor neurons in amyotrophic lateral sclerosis (Kasai et al., 2011), which is an age-related neurodegenerative disease. In addition, our recent study showed that APJ is expressed in RGCs of adult mice, and apelin deficiency increases the loss of RGCs induced by N-methyl-D-aspartate (Ishimaru et al., 2017). Recently, it was reported that the deletion of apelin or APJ in mice exhibits enhanced cardiovascular, renal, and reproductive aging (Rai et al., 2017), suggesting that the apelin-APJ system has a crucial role in cell and tissue homeostasis.

In the present study, we investigated whether endogenous apelin plays a protective role against the loss of RGC with age.

## Materials and Methods

### Mice

All mice experiments were performed in accordance with protocols approved by the Committee for the Ethical Use of Experimental Animals and the Safety Committee for Recombinant DNA Experiments at Setsunan University. Two- and 12-months-old male wild-type (WT) and apelin-knockout (KO) mice on a C57BL/6N background were used. The generation of apelin-KO mice was described previously (Kidoya et al., 2008).

### Genotyping

Five microliters of the blood sample was collected from the mouse tail vein and incubated in 50 μl of 50 mM sodium hydroxide solution for 30 min at room temperature, followed by neutralization with 5.5 μl of 1 M Tris-HCl (pH 8.0) and centrifugation to remove the insoluble fraction. Polymerase chain reaction (PCR) was performed with a Thermal Cycler T100 (BIO-RAD Laboratories, Hercules, CA, USA) and a KOD-FX DNA polymerase (TOYOBO, Osaka, Japan). Genotyping for apelin deficiency was performed by using the primers described in the previous study (Kasai et al., 2008). Reactions initially were denatured at 94°C for 2 min followed by 30 cycles at 98°C for 10 s, 70.3°C for 2 min and a final extension at 70.3°C for 2 min. Amplicons were separated using 1% agarose gel and visualized under UV light after staining with ethidium bromide. Genotyping for the rd8 mutation was performed by using the primers and methods described in the study of Mattapallil et al. (2012); however, PCR reactions were carried out for 35 cycles at 94°C for 30 s, 55.7°C for 30 s, and 72°C for 30 s, Amplicons were separated using 3% agarose gel.

### Real-Time Reverse-Transcription Polymerase Chain Reaction (RT-PCR)

Total RNA was isolated from the retinal tissues by using the SV Total RNA Isolation System (Promega, Madison, WI, USA). One microgram of total RNA was reverse transcribed with M-MLV Reverse Transcriptase (Invitrogen, Carlsbad, CA, USA) and random primers (Invitrogen). One-twentieth of the total cDNA (50 ng of equivalent RNA) was used in each amplification reaction. Real-time RT-PCR was performed with a Thermal Cycler Dice Real-Time System (Takara, Ohtsu, Japan) and THUNDERBIRD Probe qPCR Mix (TOYOBO) or THUNDERBIRD SYBR qPCR Mix (TOYOBO). The following TaqMan probe was used for real-time RT-PCR assays: apln (apelin), Mm00443562_m1; and APLNR (APJ), Mm00442191_s1 (Applied Biosystems, Foster City, CA, USA). Reactions initially were denatured at 95°C for 1 min followed by 45 cycles at 95°C for 5 s, and 60°C for 30 s, The sequences of the gene-specific primers used are as follows: 36B4 (encodes acidic ribosomal phosphoprotein PO; forward, 5′-CACTGGTCTAGGACCCGAGAAG-3′; reverse, 5′-GGTGCCTCTGGAGATTTTCG-3′) and glutamate aspartate transporter (GLAST; forward, 5′-GATCGGAAACATGAAGGAGC-3′; reverse, 5′-CAAGAAGAGGATGCCCAGAG-3′). Reactions initially were denatured at 95°C for 1 min followed by 40 cycles at 95°C for 10 s, 55°C for 20 s, and 72°C for 20 s, A melting curve analysis was carried out after amplification to verify the accuracy of the amplicon formation.

### Immunofluorescence

Mice were anesthetized with an intraperitoneal injection of a mixture of medetomidine (0.3 mg/kg), butorphanol (5 mg/kg), and midazolam (4 mg/kg) and then perfused with saline and 4% paraformaldehyde in phosphate buffer. Enucleated eyes were sequentially fixed for 24 h in 4% paraformaldehyde solution, embedded in paraffin, and cut sagittally into sections of 3-μm thickness through the cornea and parallel to the optic nerve. The antigens in the retinal sections were retrieved by microwave heating in Tris-EDTA buffer (pH 8.0) containing 0.05% Tween-20. Then the sections were exposed to Tris-buffered saline (pH 7.4) including 0.5 or 5% skim milk, 40 μg/ml Fab fragment goat anti-mouse IgG (115-007-003, Jackson ImmunoResearch Laboratories Inc., West Grove, PA, USA), and 0.5% Triton-X 100 and then incubated with mouse anti-Brn-3a (sc-8429, Santa Cruz Biotechnology Inc., Santa Cruz, CA, USA, 1:50), rabbit anti-APJ (Kasai et al., 2010; 1:50), and mouse anti-apelin (013-25871, Wako Pure Chemical Ind., Limited, Osaka, Japan, 1:100) antibodies. The secondary antibody used was biotinylated goat anti-mouse IgG antibody (E0433, DAKO Corp., Carpinteria, CA, USA, 1:500). The biotinylated antibody was detected with streptavidin-conjugated with FITC (554060, BD Biosciences, San Diego, CA, USA, 1:500) or conjugated with Alexa Fluor 568 (S11226, Invitrogen, 1:500). Anti-APJ antibody was labeled with FITC by using a fluorescein labeling kit (DOJINDO Lab, Kumamoto, Japan). Nuclei were detected with Hoechst33342 (Sigma–Aldrich, St. Louis, MO, USA, 1:1,000). Photographs were taken with a fluorescence microscope (AZ-100M, Nikon, Tokyo, Japan). The number of RGCs for each eye was determined by counting the number of Brn-3a-positive cells per retinal section and averaging results from six sections per individual.

### Electroretinography

Electroretinography was performed as previously described (Ishimaru et al., 2017). In brief, dark-adapted mice were anesthetized with a mixture of medetomidine (0.3 mg/kg), butorphanol (5 mg/kg), and midazolam (4 mg/kg), and then treated with a mydriatic agent (Mydrin P; Santen Pharmaceutical Company Limited, Osaka, Japan) and a corneal anesthetic drug (Benoxil 0.4% solution; Santen Pharmaceutical Company Limited, Osaka, Japan). A different electrode, an indifferent electrode, and a ground electrode were placed on the cornea, mouth, and tail of mice, respectively. Electroretinograms were evoked by a white light flash (3.0 × 10^−5^ cd·s/m^2^) with an LS-100 (Mayo; Nagoya, Japan), a light-emitting device, and were recorded with a corneal electrode and Powerlab data acquisition system (AD Instruments, Mountain View, CA, USA). The amplitudes of the STR were quantified by measuring from the baseline to the maximum peak of the waveforms.

### Statistics

**Figure 1:**
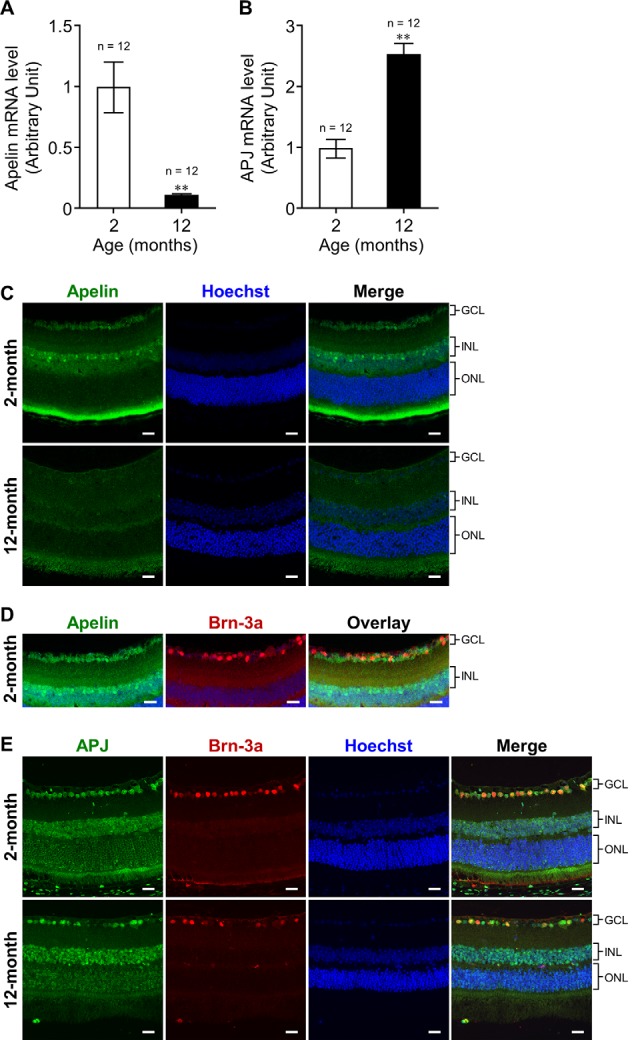
Effect of aging on the expression of apelin and APJ in the retina of mice. **(A,B)** Apelin **(A)** and APJ **(B)** mRNA expression levels in the retinas of wild-type (WT) mice at 2 and 12 months of age were assessed by real-time polymerase chain reaction (RT-PCR; *n* = 12 mice per group). 36B4 (encodes acidic ribosomal phosphoprotein PO) was used as the internal standard to normalize mRNA levels. **(C,E)** Representative images show immunofluorescence of apelin **(C)** and double immunofluorescence of APJ and Brn-3a [an Retinal ganglion cell (RGC) marker; **E**] in retinal sections from WT mice at 2 and 12 months of age. **(D)** Immunostaining of apelin and Brn-3a was performed on adjacent sections from WT mice at 2 months of age. GCL, ganglion cell layer; INL, inner nuclear layer; ONL, outer nuclear layer. Scale bars, 20 μm. Data were analyzed by unpaired student’s *t*-test and represent the mean ± SEM. **p < 0.01.

**Figure 2:**
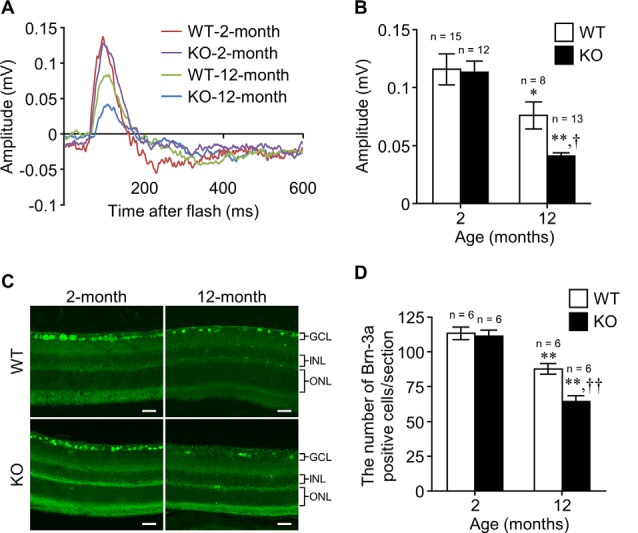
Apelin deficiency accelerates the age-related loss of RGCs in mice. **(A)** Representative waveforms show the STR evoked by 3.0 × 10^−5^ cd·s/m^2^ in the retinas of WT and apelin-knockout (KO) mice at 2 and 12 months of age. **(B)** The STR amplitudes were quantified by measuring from the baseline to the maximum peak in the waveforms (*n* = 15 mice per group for WT-2-months, *n* = 8 mice per group for WT-12-months, *n* = 12 mice per group for KO-2-months, *n* = 13 mice per group for KO-12-months). **(C)** Representative images show immunofluorescence of Brn-3a in retinal sections from WT and apelin-KO mice at 2 and 12 months of age. GCL, ganglion cell layer; INL, inner nuclear layer; ONL, outer nuclear layer. Scale bars, 20 μm. **(D)** The number of Brn-3a-positive cells per retinal section was counted (*n* = 6 mice per group). Data were analyzed by two-way ANOVA with Bonferroni’s *post hoc* test and represent the mean ± SEM. *p < 0.05 and **p < 0.01 vs. 2-months, ^†^*p* < 0.05 and ^††^*p* < 0.01 vs. WT.

Differences between groups were analyzed using unpaired student’s *t*-test (Figures 1A,B) or two-way ANOVA with Bonferroni’s *post hoc* test (Figures 2B,D). *P*-values < 0.05 were considered statistically significant. All results are expressed as the mean ± SEM.

## Results

### Influence of Aging on Apelin and APJ Expressions in the Retina

To investigate the influence of aging on apelin and APJ expression in the retina, we measured their expression levels in the retinas of 2-months- and 12-months-old mice. Real-time RT-PCR demonstrated that the expression of retinal apelin mRNA was markedly lower in the old mice than that in the young ones (Figure 1A). In contrast, retinal APJ mRNA expression was significantly higher in the old mice than in the young ones (Figure 1B). To determine apelin and APJ expressing cells, we performed immunostaining for apelin and APJ in the retina of 2-months- and 12-months-old mice. Apelin immunoreactivity was observed in cells belonging to the ganglion cell layer (GCL) and cells located in the inner part of the inner nuclear layer (INL) in the retinas of 2-months- and 12-months-old mice (Figure 1C). The apelin immunoreactivities did not show overlap with Brn-3a- (an RGC marker) positive cells in adjacent sections (Figure 1D). APJ was detected in the Brn-3a-positive RGCs of the old mice, as well as in those of the young mice (Figure 1E).

### Apelin Deficiency Accelerates the Age-Related Loss of RGCs

To examine the effect of apelin deficiency on the decline of RGCs with age, we assessed the scotopic threshold response (STR), which mainly reflects the activity of RGCs determined by electroretinography, in WT and apelin-KO mice at 2 and 12 months of age. There was no significant difference in the STR between WT and apelin-KO mice at 2 months of age (Figures 2A,B). In contrast, the STR in the apelin-KO mice at 12 months of age was markedly reduced compared with that in the age-matched WT mice (Figures 2A,B). Consistent with the results of the electroretinography, apelin deficiency enhanced the decrease in the number of Brn-3a-positive cells in the 12-months-old mice (Figures 2C,D). Eosin and Hematoxylin staining also showed that the number of the cells in the GCL was significantly decreased in aged apelin-KO mice (WT-2-months: 306.61 ± 1.93, KO-2-months: 293.11 ± 7.45, WT-12-months: 278.21 ± 1.01, KO-12-months: 246.14 ± 7.63).

## Discussion

Aging decreases the expression levels of apelin and APJ in multiple organs and tissues (Rai et al., 2017). In keeping with the report, apelin expression was markedly reduced in the retina of the old mice, indicating that senescence down-regulated the expression of apelin in the retina. Apelin immunoreactivity was detected in cells belonging to the GCL and cells located in the inner part of the INL, suggesting that the decrease of apelin expression is associated with the loss or dysfunction of cells present in the GCL and the loss of INL cells such as amacrine cells, which decrease with age-associated loss of RGCs (Samuel et al., 2011; Akopian et al., 2016). In contrast to apelin, the expression of APJ was up-regulated in the aging retina. Although we could not identify the retinal cells with increased expression of APJ with age, we showed that APJ was present in RGCs, whereas apelin was expressed in some cells surrounding RGCs in young mice. Moreover, we found that apelin deficiency in mice facilitated RGC degeneration with age in both histological and functional aspects. These results suggest that the up-regulation of APJ expression in the older mice might be a compensatory response to the decrease in apelin expression to promote RGC survival and that the apelin-APJ system contributes to RGC maintenance; however, further investigations are needed to elucidate the phenomenon.

Apelin deficiency facilitated the loss of RGCs in the old mice, but it did not affect that in the young ones, thus suggesting that chronic lack of apelin renders RGCs vulnerable to age-induced cell loss. Recent studies showed that long-term administration of apelin from periphery suppresses age-related cardiac hypertrophy and the degenerative loss of skeletal muscle (Rai et al., 2017; Vinel et al., 2018), indicating that apelin supplementation could rescue tissues and organs from senescence. In contrast to these tissues, the retina does not receive apelin from the bloodstream because of the blood-retinal barrier. Therefore, it is necessary to design APJ agonists that can cross the blood-retinal barrier and confirm their protective effect against the loss of RGCs with age in future work.

Although the data reported here provide that endogenous apelin plays a role in protecting RGCs against aging, the underlying mechanisms remain to be established. The loss of RGCs with age is considered to be induced by, at least in part, abnormal glutamate metabolism (Henneberry et al., 1989). Indeed, the expression of GLAST, which is a glutamate transporter expressed in Müller cells and regulates the synaptic activity in the inner retina, was markedly decreased in the aging retina (Young, 1.00 ± 0.08; Old, 0.14 ± 0.02). Our previous study showed that RGCs of apelin-KO mice are vulnerable to glutamate-induced excitotoxicity (Ishimaru et al., 2017). Therefore, endogenous apelin might protect against the age-related loss of RGCs by suppressing glutamate excitotoxicity induced by the decline of glutamate uptake into Müller cells. However, further investigations are required, given the role of apelin on oxidative stress, autophagy (Foroughi et al., 2019), and mitochondrial dysfunction (Zeng et al., 2010), all of which are associated with the degeneration of RGCs with age (Militante and Lombardini, 2004; Chrysostomou et al., 2010; Boya, 2017).

In the present study, we used apelin-KO mice and their WT controls mice maintained on a C57BL/6N background. C57BL/6N mice are a common strain used worldwide for the creation of single-gene KOs, but the mice carry an rd8 mutation and several ocular abnormalities (Mattapallil et al., 2012; Moore et al., 2018). We examined the rd8 mutation in our mice and observed the presence of the rd8 mutation in homozygous form in both WT mice and apelin-KO mice (Supplementary Figure S1). Although the aforementioned reports revealed that the rd8 mutation causes degeneration in the inner and outer nuclear layers, the outer plexiform layer, as well as the photoreceptor outer segments, there was no obvious change in the ganglion cell layer in C57BL/6N mice with or without the rd8 mutation. In addition, electroretinography in the rd8 mutation mice remains relatively stable for 1 year (Chang et al., 2002). Therefore, our data, taken together with others, imply that the accelerated degeneration of RGCs in apelin-KO mice at 12 months of age was due to apelin deficiency but not the rd8 mutation, it is necessary, however, to examine the effect of apelin deficiency on the aging retina under conditions of the rd8-free background.

In conclusion, to our knowledge, this is the first report showing that endogenous apelin plays a protective role in retinal tissue during aging and that the apelin-APJ system may be a new target for preventing age-related degeneration of RGCs; however, further studies, including the effect of APJ-KO and intravitreal injection of apelin on the aging retina, are necessary to determine the precise role of the apelin-APJ system on the loss of RGCs with age.

## Data Availability Statement

The datasets generated for this study are available on request to the corresponding author.

## Ethics Statement

The animal study was reviewed and approved by the Committee for the Ethical Use of Experimental Animals and the Safety Committee for Recombinant DNA Experiments at Setsunan University. Written informed consent was obtained from the owners for the participation of their animals in this study.

## Author Contributions

YI conceived the study and wrote the manuscript. YI, AS, and FS conducted the experiments and analyzed the data. YI, AS, FS, AY, YY, and SM interpreted the results. SM assisted in the manuscript preparation.

## Conflict of Interest

The authors declare that the research was conducted in the absence of any commercial or financial relationships that could be construed as a potential conflict of interest.
