# Supplementary material for: Endogenous Apelin Is Protective Against Age-Associated Loss of Retinal Ganglion Cells in Mice
Source: Front Aging Neurosci. 2020 Mar 20;12:58. doi: 10.3389/fnagi.2020.00058 (PMC7141441; doi:10.3389/fnagi.2020.00058)
Supplement: Supplementary file 1 [file Data_Sheet_1.PDF]

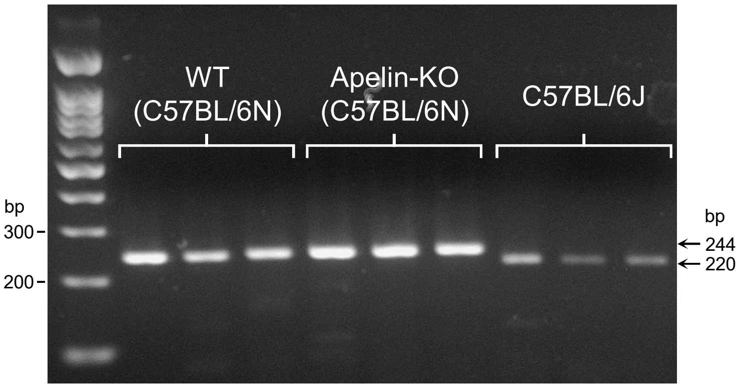

Supplementary Figure 1. Genotyping for the rd8 mutation. The rd8 mutation in WT and apelin-KO mice was determined by PCR (see Material and Methods for details). DNA samples from C57BL/6J mice were used as a negative control for the rd8 mutation. rd8 mutation homozygous, 244 bp; rd8-free, 220 bp
